# Burrow collapse is not the only explanation for rapid, noncatastrophic preservation of 3D dinosaurs

**DOI:** 10.1073/pnas.2423019122

**Published:** 2025-01-09

**Authors:** Meaghan M. Emery-Wetherell

**Affiliations:** ^a^College of Information Science, University of Arizona Tucson, AZ 85721

MacLennan et al. (1) report new radiometric dates and sedimentological context for the exceptionally preserved dinosaurs in the lower Yixian Formation. However, the sedimentology and provenance analysis provided do not support the authors’ interpretation of preservation exclusively via burrow collapses.

The authors conclude that the sediment gradient between the body cavity and its exterior suggests a sieving effect as the bodies rotted (1). Yet as described, this sieving is the result of a fresh body being covered in sediment, which is not exclusive to burrows. It requires a recent death prior to a geologic event, which would also be accounted for by lahars or flood events as suggested by previous work (2, 3).

The authors found older detrital zircons in both *Psittacosaurus* specimens, most likely from posteruptive mixing with material on the landscape (1). This is evidence of mobilization of the volcaniclastic material, which does not exclude a flood or lahar origin. Lahars often incorporate sediment along their pathway, particularly when associated with large-scale mass wasting events (4).

There also remain undiscussed contradictions with the burrow hypothesis, including that burrow collapse is not necessarily fatal for modern animals. MacLennan et al. (1) cited evidence for high mortality in penguins, but this is a problem for penguin chicks, not adults (5). Survival rates also increase dramatically at 3 wks of age when chicks can dig themselves out (5). Other animals like tortoises survive and escape even when their burrows are collapsed by heavy machinery (6).

Death by burrow collapse was suggested as an explanation for why the dinosaurs are preserved at rest without evidence of pain or struggle. But to do so, a burrow collapse would need to be catastrophic enough to either instantly kill or inextricably pin the animal in place. Both scenarios would necessitate considerable pressure from a collapsed roof.

The Lujiatun fossils lack the expected osteological and postural signs of crushing or compression. Furthermore, many show signs of postmortem movement inconsistent with complete entombment, including full-body imbrication (2, 7) and limb entanglement (8).

Burial of recently dead animals by a low-force flood or lahar would explain the sediment gradient and zircon provenance described by the authors (1) as well as provide imbrication. An abundance of recently dead animals can be provided by volcanic gassing, particularly CO_2_ poisoning which is known to kill people without waking them (9). In fact, CO_2_ typically is strongest near the ground (10), which may explain why the primary natural posture at Lujiatun is of sleep rather than active movement.

Both this ref. 1 and other work ref. 3 have concluded that Lujiatun does not represent a single mortality event. But CO_2_ poisoning can be a regular occurrence in volcanically active areas like the Goma region of Africa, which hosts near-permanent CO_2_ vents or *mazuku* (10). *Mazuku* kill people annually and can be identified by their regular accumulation of dead animals (10). Suffocation by volcanic gases should be considered as an important contributor to normal attrition in volcanic regions.

## References

[1] S. A. MacLennan , Extremely rapid, yet noncatastrophic, preservation of the flattened-feathered and 3D dinosaurs of the Early Cretaceous of China. Proc. Natl. Acad. Sci. U.S.A. **121**, e2322875121 (2024). 39495941 10.1073/pnas.2322875121 PMC11588062

[2] Z. Qi, P. M. Barrett, D. A. Eberth, Social behaviour and mass mortality in the basal ceratopsian dinosaur *Psittacosaurus* (Early Cretaceous, People’s Republic of China). Palaeontology **50**, 1023–1029 (2007).

[3] C. S. Rogers , The Chinese Pompeii? Death and destruction of dinosaurs in the Early Cretaceous of Lujiatun, NE China. Palaeogeogr. Palaeoclimatol. Palaeoecol. **427**, 89–99 (2015).

[4] A. Encinas , Pliocene lahar deposits in the Coastal Cordillera of central Chile: Implications for uplift, avalanche deposits, and porphyry copper systems in the Main Andean Cordillera. J. South Am. Earth Sci. **20**, 369–381 (2006).

[5] P. J. Seddon, Y. van Heezik, Effects of hatching order, sibling asymmetries, and nest site on survival analysis of Jackass Penguin chicks. Auk **108**, 548–555 (1991).

[6] M. Mendonca, R. Beauman, H. E. Balbach, Burrow Collapse as a Potential Stressor on the Gopher Tortoise (US Army Corps of Engineers, 2007).

[7] B. P. Hedrick , The osteology and taphonomy of a *Psittacosaurus* bonebed assemblage of the Yixian Formation (Lower Cretaceous), Liaoning, China. Cret. Res. **51**, 321–340 (2014).

[8] G. Han , An extraordinary fossil captures the struggle for existence during the Mesozoic. Sci. Rep. **13**, 11221 (2023). 37464026 10.1038/s41598-023-37545-8 PMC10354204

[9] G. N. Wagner, M. A. Clark, E. J. Koenigsberg, S. J. Decata, Medical evaluation of the victims of the 1986 Lake Nyos disaster. J. Forensic Sci. **33**, 899–909 (1988). 3139823

[10] B. Smets , Dry gas vents (“mazuku”) in Goma region (North-Kivu, Democratic Republic of Congo): Formation and risk assessment. J. Afr. Earth Sci. **58**, 787–798 (2010).

